# Supplementary material for: Adaptation to Virtual Assessment during the COVID-19 Pandemic: Clinical Case Presentation Examination
Source: Dent J (Basel). 2023 Feb 9;11(2):45. doi: 10.3390/dj11020045 (PMC9955899; doi:10.3390/dj11020045)
Supplement: Supplementary file 1 [file dentistry-11-00045-s001.zip › dentistry-2003642-supplementary.pdf]

dentistry journal

Supplementary File 1

Case Presentation Questions

Consent section

I confirm that I have read and understood the Participant Information Sheet version 1.0 dated 07th February 2022
I confirm that I have read and understood the University of Glasgow Student Privacy Notice (found here—https://www.gla.ac.uk/myglasgow/dpfoioffice/gdpr/privacynotices/studentprivacynotice/)
I have had the opportunity to think about the information and ask questions and understand the answers I have been given.
I understand that my participation is voluntary and that I am free to withdraw at any time without giving any reason and without my legal rights being affected. However, after completing the surveys, I understand that as the data will be fully anonymised, it will not be possible to withdraw my responses.
I confirm that I agree to the way my data will be collected and processed and that data will be stored for up to 10 years in University archiving facilities in accordance with relevant Data Protection policies and regulations.
I understand that all data and information I provide will be kept confidential and will be seen only by study researchers and regulators whose job it is to check the work of researchers.
I agree to take part in the study.

1. Do you consent to all the points above and agree to participate in this study?
   - O Yes
   - O No

2 Prior to the online examination I was confident it could take place online
   - O Strongly Agree
   - O Agree
   - O Neither Agree nor Disagree
   - O Disagree
   - O Strongly Disagree

3 I was given enough information to allow me to prepare adequately for the online examination
   - O Strongly Agree
   - O Agree
   - O Neither Agree nor Disagree
   - O Disagree
   - O Strongly Disagree

4 Preparation of the PowerPoint for the online examination was much more time consuming than preparation for the face-to-face examination
   - O Strongly Agree
   - O Agree
   - O Neither Agree nor Disagree
   - O Disagree
   - O Strongly Disagree

5 The IT used in the online examination worked well
   - O Strongly Agree
   - O Agree
   - O Neither Agree nor Disagree
   - O Disagree
   - O Strongly Disagree

6 Interaction with the examiners online was more difficult than in the face-to-face examination

- O Strongly Agree
- O Agree
- O Neither Agree nor Disagree
- O Disagree
- O Strongly Disagree

7 My ability to demonstrate my knowledge online was the same as in the face-to-face examination

- O Strongly Agree
- O Agree
- O Neither Agree nor Disagree
- O Disagree
- O Strongly Disagree

8 The face-to-face examination was fairer to the student sitting the examination

- O Strongly Agree
- O Agree
- O Neither Agree nor Disagree
- O Disagree
- O Strongly Disagree

9 It was more stressful sitting the exam face-to-face than online

- O Strongly Agree
- O Agree
- O Neither Agree nor Disagree
- O Disagree
- O Strongly Disagree

10 Following the online examination, I would be confident that case presentation could be examined online in future.

- O Strongly Agree
- O Agree
- O Neither Agree nor Disagree
- O Disagree
- O Strongly Disagree

11 I would prefer to sit this examination online.

YES ☐

NO ☐

If you answer No, please go to Q 11

If you answer Yes, please go to Q 12

11. If you preferred to sit the exam online, what were your reasons?

12. If you would have preferred to sit the exam face-to-face, what were your reasons?

13. In the box below, list THREE words that describe the online examination experience for you. (e.g., *simple, impossible, easy, difficult, boring, unfair, etc.)*

14. What, if any, were the most difficult parts of the online examination process?

15. What, if any, are the improvements that should be made to the online examination process?
